# Sexual behaviors and associated factors among first-year undergraduates at overseas Chinese-oriented universities in China

**DOI:** 10.3389/fpubh.2025.1609949

**Published:** 2025-08-22

**Authors:** Zulin Chen, Yikun Zheng, Lihan Lin, Yongjun Chen, Yunting Zheng, Hongmiao Chen

**Affiliations:** ^1^School of Physical Education, Huaqiao University, Quanzhou, China; ^2^Research Center for Sports and Health Sciences, Huaqiao University, Quanzhou, China; ^3^Provincial University Key Laboratory of Sport and Health Science, School of Physical Education and Sport Science, Fujian Normal University, Fuzhou, China; ^4^Quanzhou Center for Disease Control and Prevention, Quanzhou, China; ^5^School of Health Management, Fujian Medical University, Fuzhou, China

**Keywords:** overseas Chinese-oriented universities, first-year students, sexual behavior, HIV/AIDS, Monkeypox

## Abstract

**Background:**

This study aims to analyze the status of sexual behavior and its influencing factors among first-year undergraduate students in Chinese overseas Chinese schools, providing scientific evidence for university sexual health education and public health interventions. It also explores sexual behavior patterns and prevention strategies in a multicultural context.

**Methods:**

A cluster sampling method was employed to conduct a questionnaire survey among the 2024 cohort of freshmen at Huaqiao University (*n* = 4,892) in October 2024. The questionnaire covered basic sociodemographic characteristics, knowledge about HIV and Monkeypox prevention, and sexual behavior. Chi-square tests and binary logistic regression models were used to analyze differences in sexual behavior among students with different characteristics and main influencing factors.

**Results:**

The study found that the sexual behavior occurrence rate among students was 6.6%. Sensitivity analyses were conducted to verify the robustness of key associations. The rate was significantly higher among students from Hong Kong, Macau, and Taiwan compared to those from Mainland China and abroad (*p* < 0.05), which was corroborated by the logistic regression (OR = 3.737, 95% CI: 2.846–4.908). Students with higher knowledge about Monkeypox had a higher occurrence of sexual behavior (OR = 1.174, 95% CI: 1.060–1.301). Students who were aware of the consequences of homosexual behavior had a significantly lower occurrence of sexual behavior (OR = 0.453, 95% CI: 0.346–0.594). Compared with those who opposed homosexual behavior, students with neutral and supportive attitudes toward homosexual behavior had a lower occurrence of sexual behavior (OR = 0.377, 95% CI: 0.287–0.496; OR = 0.371, 95% CI: 0.218–0.629). Students who received HIV prevention services in the past year had a lower occurrence of sexual behavior (OR = 0.294, 95% CI: 0.213–0.405). In comparison, those who participated in HIV awareness services in the past year had a higher occurrence of sexual behavior (OR = 4.280, 95% CI: 3.140–5.834). All associations remained robust in sensitivity analyses.

**Conclusion:**

The occurrence rate of sexual behavior among first-year students in Chinese overseas Chinese schools is relatively low, but significant differences are observed based on region of origin, gender, and knowledge level. The study indicates that sexual health education should focus on students’ cultural background, gender roles, and cognitive differences, combining behavioral interventions to enhance the relevance and effectiveness of education and reduce high-risk sexual behaviors.

## 1 Introduction

With the evolution of social norms and the accelerating internationalization of higher education, sexual behaviors among university students have emerged as a critical public health research priority (1). As a key demographic in tertiary institutions, first-year undergraduates’ knowledge and behaviors are predominantly shaped by pre-university education (2). Their sexual health literacy and behavioral patterns not only reflect individual health status but are also profoundly influenced by multifaceted factors, including cultural backgrounds of origin, educational environments, and societal perceptions (3). This dynamic is particularly pronounced in Chinese overseas Chinese-oriented universities, where student population diversity and unique cultural contexts may engender distinct patterns of sexual behavior development compared to conventional universities (4, 5).

The global resurgence of sexually transmitted infections (STIs), notably Human Immunodeficiency Virus/Acquired Immunodeficiency Syndrome (HIV/AIDS) and Monkeypox, has further underscored the urgency of investigating sexual behaviors in collegiate populations (6, 7). High-risk sexual practices among youth significantly amplify HIV transmission risks, with insufficient sexual health knowledge and inconsistent protective measures being primary vulnerability factors in this cohort (7). Concurrently, Monkeypox outbreaks have been epidemiologically linked to multi-partner sexual networks and inadequate protective behaviors, particularly among socially active groups like university students (8). These developments highlight the imperative need to elucidate university students’ sexual behavior patterns and their determinants for effective STI prevention (9).

Prior research has identified significant variations in sexual behaviors across sociodemographic dimensions among university students while demonstrating the moderating effects of sexual health education and targeted interventions on behavioral outcomes (10). Nevertheless, systematic investigations into sexual behavior determinants among first-year students in overseas Chinese-oriented universities remain scarce. The unique cross-cultural exposures and institutional environments characterizing these students may engender distinctive mechanisms shaping sexual health cognition and practices, presenting a critical research opportunity (11).

This study aims to systematically analyze the current status and factors influencing sexual behaviors among first-year undergraduates in Chinese overseas Chinese-oriented universities. Data were collected using a structured, self-administered questionnaire among all first-year students at Huaqiao University in October 2024. The survey included questions on sociodemographic characteristics, knowledge of HIV and Monkeypox prevention, and sexual behaviors. The findings will provide evidence-based recommendations for campus-based sexual health education and public health interventions while contributing novel theoretical and practical insights to research on sexual behaviors in multicultural academic settings.

## 2 Methods

### 2.1 Study design and survey participants

#### 2.1.1 Study participants

Huaqiao University was chosen for its unparalleled demographic diversity, with over 40% of its student body comprising overseas Chinese and individuals from more than 90 countries, creating a natural laboratory to examine how pre-existing cultural norms interact with acculturative influences on sexual behavior. This institution’s role as a global hub for diasporic communities ensures findings transcend localized contexts, offering actionable insights for both Chinese and international public health strategies targeting culturally heterogeneous youth populations. In October 2024, a cross-sectional survey was conducted among all first-year undergraduates enrolled at Huaqiao University’s Quanzhou and Xiamen campuses. Inclusion criteria were: (1) Enrolled as first-year undergraduates in 2024; (2) aged 18–24 years; (3) no visual impairments; (4) absence of severe mental health conditions; (5) capability to independently complete paper-based questionnaires; (6) voluntarily provided informed consent.

### 2.2 Study design

#### 2.2.1 Sampling method

Cluster sampling was used, treating academic classes as the cluster units. All first-year undergraduate classes matriculating in September 2023 at Huaqiao University were included, effectively constituting a census of the target population. Because every eligible cluster was retained, no random, stratified, or convenience selection was required. All students within each cluster were invited to participate, and no within-cluster subsampling was performed. This comprehensive inclusion minimized selection bias and maximized sample representativeness.

#### 2.2.2 Data collection

A self-administered paper questionnaire was distributed and collected on-site. The questionnaire was adapted from the *University Students’ HIV Prevention Knowledge and Competency Assessment Questionnaire* (12) and updated to align with global advancements in HIV/AIDS and Monkeypox prevention. The questionnaire assessed: (1) Sociodemographic characteristics included sex and place of origin, both treated as nominal categorical variables; age was applied only as an inclusion criterion, was not recorded precisely, and therefore could not be analyzed. (2) Knowledge of HIV/AIDS and Monkeypox prevention (12 items in total), consisting of eight nationally endorsed HIV items and four investigator-developed Monkeypox items; each item was scored 1 for a correct response and 0 for an incorrect or missing response, and the subsection totals (0–8 for HIV, 0–4 for Monkeypox) were analyzed as continuous composite variables. (3) Sexual-behavior variables: sexual experience (binary); stage of sexual debut (ordinal categorical); partner type at debut (nominal categorical); condom use at first intercourse (nominal categorical); commercial-sex engagement (binary); condom use at last commercial encounter (nominal categorical); frequency of condom use in commercial sex (ordinal categorical); attitude toward same-sex relationships (ordinal); awareness of consequences (binary); receipt of HIV-prevention education (binary); participation as a campaign facilitator (binary). The study protocol was approved by the Quanzhou Center for Disease Control and Prevention Ethics Committee (No. 2024003), and all respondents were required to provide written informed consent.

#### 2.2.3 Quality control

(1) Questionnaire validation: Content validity was ensured through expert consultations (HIV/AIDS, Monkeypox, and sexual behavior specialists) and a pilot survey (July–September 2024) to refine clarity and feasibility. (2) Bilingual adaptation: Professionally translated English questionnaires were reviewed by experts to eliminate ambiguities for participants with limited Chinese proficiency. (3) Data collection rigor: Trained investigators distributed paper questionnaires on-site, provided real-time clarification, and ensured immediate return to minimize non-response and data loss. Anonymous submission safeguarded participant privacy. (4) Data verification: A dedicated quality control team cross-validated all entries.

#### 2.2.4 Data analysis

Data were analyzed in SPSS 26.0 after rigorous cleaning. Continuous variables are summarized as median and inter-quartile range (IQR), and categorical variables as frequencies (%). Univariate analyses of associations between independent variables and sexual behavior were conducted using *χ*^2^ tests and univariate binary logistic regression, with results reported as crude odds ratios (ORs) and 95% confidence intervals (CIs) calculated as OR = *e^β^*. Variables with *p* < 0.05 in univariate analyses were entered into a binary logistic regression analysis with sexual activity status (0 = no, 1 = yes) as the dependent variable, using the enter method to estimate adjusted ORs and 95% CIs. Statistical significance was set at *α* = 0.05.

In addition to the primary analysis, several sensitivity checks were performed to validate model assumptions and robustness. The Box–Tidwell procedure was used to examine the linearity-in-the-logit assumption for the two continuous composite predictors (HIV Knowledge Score and Monkeypox Knowledge Score). For the ordinal predictor Attitude toward same-sex sexual behavior, we conducted a linear-by-linear trend chi-square test and also re-ran the logistic regression treating this three-level attitude variable as a continuous predictor (coded 1 to 2 to 3) to assess the consistency of its effect. Region of origin, a nominal categorical variable with multiple groups, was excluded from these sensitivity analyses because it lacks an inherent ordinal scale for trend or log-linearity testing.

## 3 Results

### 3.1 Participant characteristics

A total of 5,341 questionnaires were distributed, with 5,310 returned (response rate 99.4%). After data cleaning and exclusion of 418 invalid responses (e.g., incomplete or inconsistent entries), 4,892 valid questionnaires were retained, resulting in a valid response rate of 92.1%. The sample comprised 2,615 male students (53.5%) and 2,277 female students (46.5%). In terms of geographic origin, 3,973 participants (81.2%) were from mainland China, 753 (15.4%) from Hong Kong, Macao, and Taiwan (HMT), and 166 (3.4%) were international students, indicating a predominance of mainland Chinese students followed by HMT (Hong Kong, Macau, and Taiwan) and international cohorts.

### 3.2 Sexual behavior patterns among first-year undergraduates

#### 3.2.1 General sexual behavior profile

The survey revealed that 4,567 students (93.4%) reported no prior sexual experience, while 325 students (6.6%) indicated having engaged in sexual activity, demonstrating that the vast majority remained sexually inexperienced (Table 1).

**Table 1:** Differences in sexual behavior among undergraduate first-year students with different population and sociological characteristics [*n* (%), *n* = 4,892].

Survey item	Sexual behavior		*χ* ^2^	*p*-value
	Yes	No		
Gender			62.344	<0.001
Male	218 (8.3)	2,397 (91.7)		
Female	107 (4.7)	2,170 (95.3)		
Region of origin			127.456	<0.001
Mainland China	179 (4.5)	3,794 (95.5)		
HMT	131 (17.4)	622 (82.6)		
International	15 (9.0)	151 (91.0)		
Total	325 (6.6)	4,567 (93.4)		

Regarding the age of sexual debut, Mainland Chinese and HMT students predominantly initiated sexual activity during high school (50.8% and 49.6%, respectively). In contrast, international students primarily reported first sexual experiences during university (66.7%). When examining partners at sexual debut, most students engaged with heterosexual partners. Notably, international students showed the highest proportion of homosexual partner selection (26.7%). Condom usage during first sexual intercourse varied by region: Mainland Chinese students reported 62.6% usage, HMT students 61.8%, while international students demonstrated significantly lower utilization rates at 46.7%. Analysis of commercial sexual activity within the past year revealed comparable rates between Mainland Chinese (16.2%) and HMT students (16.0%), with international students reporting slightly lower frequency (13.3%) (Table 2).

**Table 2:** Sexual behavior characteristics of undergraduate first-year students [*n* (%), *n* = 325].

Survey item	Region of origin		
	Mainland China (*n* = 179)	HMT (*n* = 131)	International (*n* = 15)
Educational stage at sexual debut			
≤Primary school	12 (6.7)	7 (5.3)	1 (6.7)
Junior high school	10 (5.6)	9 (6.9)	0 (0.0)
Senior high school	91 (50.8)	65 (49.6)	4 (26.7)
University	66 (36.9)	50 (38.2)	10 (66.7)
Partner type at sexual debut			
Heterosexual partner	150 (83.8)	115 (87.8)	11 (73.3)
Homosexual partner	26 (14.5)	13 (9.9)	4 (26.7)
Other	3 (1.7)	3 (2.3)	0 (0.0)
Condom use at first intercourse			
Yes	112 (62.6)	81 (61.8)	7 (46.7)
No	50 (27.9)	40 (30.5)	8 (53.3)
Uncertain	17 (9.5)	10 (7.6)	0 (0.0)
Commercial sexual activity in the past year			
Yes	29 (16.2)	21 (16.0)	2 (13.3)
No	68 (38.0)	45 (34.4)	3 (20.0)
Uncertain	82 (45.8)	65 (49.6)	10 (66.7)

#### 3.2.2 Characteristics of commercial sexual activity among first-year university students

Survey results indicated that a total of 52 first-year university students engaged in commercial sexual activity. Analysis of protective practices during commercial sexual encounters revealed significant regional variations. During their most recent commercial sexual activity, condom usage rates were 20.7% among Mainland Chinese students, 23.8% among HMT students, and 50.0% among international students.

More concerning were the patterns observed over the past year. The proportion of students reporting never using condoms was alarmingly high: 57.1% in HMT students, 37.9% in Mainland Chinese students, and 50.0% among international students. Conversely, consistent condom use (defined as use during every encounter) remained critically low: 6.9% (Mainland Chinese), 4.8% (HMT), and 0.0% (international students) (Table 3).

**Table 3:** Characteristics of commercial sexual behavior among undergraduate first-year students [*n* (%), *n* = 52].

Survey item	Region of origin		
	Mainland China (*n* = 29)	HMT (*n* = 21)	International (*n* = 2)
Condom use during most recent commercial sexual encounter			
Yes	6 (20.7)	5 (23.8)	1 (50.0)
No	7 (24.1)	7 (33.3)	1 (50.0)
Uncertain	16 (55.2)	9 (42.9)	0 (0.0)
Frequency of condom use during commercial sexual activity in the past year			
Never	11 (37.9)	12 (57.1)	1 (50.0)
Occasionally	8 (27.6)	3 (14.3)	1 (50.0)
Consistently	9 (31.0)	2 (9.5)	0 (0.0)
Uncertain	1 (3.4)	4 (19.0)	0 (0.0)

### 3.3 Disparities in sexual behavior frequency among first-year university students

#### 3.3.1 Variations by sociodemographic characteristics

Significant gender-based disparities were observed, with male students demonstrating a higher frequency of sexual activity (8.3%) compared to their female counterparts (4.7%) (*χ*^2^ = 62.344, *p* < 0.05).

Regional analysis revealed pronounced differences: HMT students exhibited the highest sexual activity rate at 17.4%, significantly exceeding rates among Mainland Chinese (4.5%) and international students (9.0%) (*χ*^2^ = 127.456, *p* < 0.05) (Table 1).

#### 3.3.2 Disparities in sexual behavior by HIV/Monkeypox knowledge levels

Both composite scores were non-normally distributed; results are therefore summarized as median (IQR). Among 4,892 respondents, the HIV Knowledge Score had a median of 7.0 with an IQR of 6.0–8.0 on the 0–8 scale, whereas the Monkeypox Knowledge Score had a median of 3.0 with an IQR of 1.0–3.0 on the 0–4 scale. In univariate logistic regression, higher HIV knowledge was inversely associated with sexual activity (OR = 0.852, 95% CI: 0.794–0.913), while higher Monkeypox knowledge was positively associated (OR = 1.122, 95% CI: 1.025–1.229) (Table 4).

**Table 4:** Differences in sexual behavior among undergraduate first-year students with different levels of knowledge about AIDS and monkeypox.

Predictor variable	*β*	S.E.	Wald *χ*^2^	*p*-value	OR	95% CI
HIV knowledge score	−0.16	0.036	20.391	<0.001	0.852	0.794–0.913
Monkeypox knowledge score	0.115	0.046	6.213	0.013	1.122	1.025–1.229

#### 3.3.3 Disparities in sexual behavior by awareness and attitudes toward same-sex sexual behavior

Students who lacked awareness of same-sex sexual behavior exhibited a significantly higher frequency of sexual activity (12.4%) compared to those with prior awareness (5.1%) (*χ*^2^ = 71.673, *p* < 0.001).

Attitudinal differences further revealed distinct patterns: Students holding opposing views toward same-sex sexual behavior demonstrated the highest sexual activity rate (12.6%), surpassing rates among those with neutral (4.0%) or supportive attitudes (4.3%) (*χ*^2^ = 121.027, *p* < 0.001) (Table 5).

**Table 5:** Differences in sexual behavior among undergraduate first-year students with different same-sex views [*n* (%), *n* = 4,892].

Survey item	Sexual behavior		*χ* ^2^	*p*-value
	Yes	No		
Awareness of potential consequences following same-sex sexual behavior			71.673	<0.001
Yes	194 (5.1)	3,640 (94.9)		
No	131 (12.4)	927 (87.6)		
Attitudes toward same-sex sexual behavior			121.027	<0.001
Opposed	187 (12.6)	1,301 (87.4)		
Neutral	119 (4.0)	2,840 (96.0)		
Supportive	19 (4.3)	426 (95.7)		

#### 3.3.4 Differences in sexual behavior among undergraduate first-year students with different AIDS health education acceptance

Survey results indicated that first-year students who did not receive HIV-prevention education in the past year reported a significantly higher frequency of sexual activity (10.5%) than those who did receive such education (5.3%) (*χ*^2^ = 39.762, *p* < 0.001). In addition, students who served as campaign facilitators for HIV-prevention activities showed a higher frequency of sexual activity (9.9%) compared with non-participants (4.0%) (*χ*^2^ = 66.396, *p* < 0.001) (Table 6).

**Table 6:** Differences in sexual behavior among undergraduate first-year students with different AIDS health education acceptance [*n* (%), *n* = 4,892].

Survey item	Sexual behavior		*χ* ^2^	*p*-value
	Yes	No		
Received HIV prevention education			39.762	<0.001
Yes	195 (5.3)	3,459 (94.7)		
No	130 (10.5)	1,108 (89.5)		
Served as campaign facilitator of HIV prevention education			66.396	<0.001
Yes	216 (9.9)	1973 (90.1)		
No	109 (4.0)	2,594 (96.0)		

#### 3.3.5 Predictors of sexual activity among first-year university students

The results demonstrated several significant associations. Compared to Mainland Chinese students, those from Hong Kong, Macau, and Taiwan (HMT) showed 3.737 times higher odds of sexual activity (95% CI: 2.846–4.908). Higher knowledge levels about Monkeypox were positively associated with sexual activity (OR = 1.174, 95% CI: 1.060–1.301), while awareness of potential consequences of same-sex sexual behavior was protective (OR = 0.453, 95% CI: 0.346–0.594). Students with neutral (OR = 0.377, 95% CI: 0.287–0.496) or supportive (OR = 0.371, 95% CI: 0.218–0.629) attitudes toward same-sex behavior had lower odds of sexual activity compared to those with opposing views. Notably, while receiving HIV prevention education was associated with reduced odds of sexual activity (OR = 0.294, 95% CI: 0.213–0.405), students who participated as campaign facilitators showed substantially higher odds (OR = 4.280, 95% CI: 3.140–5.834) (Table 7).

**Table 7:** Binary logistic regression analysis of influencing factors of sexual behavior among undergraduate first-year students.

Predictor variable	*β*	S.E.	Wald *χ*^2^	*p*-value	OR	95% CI
Gender						
Male	–	–	–	–	–	–
Female	−0.195	0.144	1.833	0.176	0.823	0.621–1.091
Region of origin						
Mainland China	–	–	–	–	–	–
HMT	1.318	0.139	89.923	<0.001	3.737	2.846–4.908
International	0.496	0.311	2.541	0.111	1.642	0.892–3.023
HIV Knowledge Score	−0.053	0.045	1.341	0.247	0.949	0.868–1.037
Monkeypox knowledge score	0.16	0.052	9.42	0.002	1.174	1.060–1.301
Awareness of potential consequences following same-sex sexual behavior						
No	–	–	–	–	–	–
Yes	−0.791	0.137	33.101	<0.001	0.453	0.346–0.594
Attitudes toward same-sex sexual behavior						
Opposed	–	–	–	–	–	–
Neutral	−0.975	0.14	48.5	<0.001	0.377	0.287–0.496
Supportive	−0.993	0.27	13.502	<0.001	0.371	0.218–0.629
Received HIV prevention education						
No	–	–	–	–	–	–
Yes	−1.225	0.163	56.366	<0.001	0.294	0.213–0.405
Served as campaign facilitator of HIV prevention education						
No	–	–	–	–	–	–
Yes	1.454	0.158	84.6	<0.001	4.28	3.140–5.834

#### 3.3.6 Sensitivity analyses

In the supplementary sensitivity analyses, all key non-binary predictors deviated significantly from normality (Shapiro–Wilk tests, *p* < 0.001 for HIV Knowledge Score, Attitude toward same-sex sexual behavior, and Monkeypox Knowledge Score; Supplementary Table S1). The Box–Tidwell tests confirmed that the logit-linearity assumption was satisfied for both continuous knowledge scores: the interaction terms for HIV Knowledge Score and Monkeypox Knowledge Score were non-significant (Wald *χ*^2^ = 0.496, *p* = 0.481; and 2.229, *p* = 0.135, respectively; Supplementary Table S2). Likewise, treating Attitude toward same-sex sexual behavior as an ordinal or continuous predictor yielded results consistent with the main analysis: a linear-by-linear trend test showed a highly significant association (*χ*^2^ = 91.817, *p* < 0.001), and coding this attitude variable as a continuous covariate in the logistic model produced an adjusted OR of 0.467 (95% CI: 0.370–0.590, *p* < 0.001), indicating a robust protective effect (Supplementary Table S3).

Taken together, the normality checks, Box–Tidwell tests, and trend analyses demonstrate that the chosen logistic-regression specification is statistically appropriate: the two knowledge scores perform well as linear continuous terms, and the attitude variable retains a stable effect when treated either categorically or as an ordinal predictor, confirming that our main findings are not sensitive to alternative model parameterisations.

## 4 Discussion

The findings of this study indicate that most university freshmen engage in limited sexual activity upon entering higher education. The overall frequency of sexual behavior among first-year undergraduates in Chinese overseas Chinese-affiliated universities was relatively low (6.6%), though higher than the 3.48% reported in a 2021 survey of freshmen from five mainland Chinese universities by Yang et al. (13). Notably, significant regional disparities were observed in sexual behavior frequency. Students from HMT exhibited markedly higher rates of sexual activity compared to their mainland Chinese and international counterparts, likely attributable to more open sexual attitudes and lesser influence from restrictive familial or sociocultural norms in these regions (14).

Variations were also evident in the timing of first sexual experiences and safety practices. Mainland Chinese and HMT students predominantly initiated sexual activity during high school, whereas international students tended to engage in first sexual encounters during university. These differences may reflect divergences in sexual norms, cultural environments, and educational systems across regions (15, 16). Additionally, international students reported a higher proportion of first sexual experiences with same-sex partners, potentially mirroring greater societal acceptance of diverse sexual orientations in their home countries (17). Despite relatively high overall condom use rates among mainland Chinese and HMT students, a substantial proportion across all regions neglected condom use during their first sexual encounter—a trend consistent with global adolescent populations (18, 19). Low consistent condom use during commercial sex across regions further highlights persistent gaps in risk awareness, cultural taboos, and economic constraints, underscoring the urgent need for enhanced sexual education and targeted interventions for high-risk behaviors (20–22).

Knowledge levels of HIV/AIDS and Monkeypox (formerly monkeypox) emerged as significant predictors of sexual behavior. Students with higher HIV/AIDS knowledge demonstrated significantly reduced sexual activity, emphasizing the protective role of sexual health literacy in mitigating high-risk behaviors (23). Conversely, greater Monkeypox knowledge correlated with increased sexual activity, possibly reflecting insufficient global public health messaging on Monkeypox risks and low perceived threat among informed individuals (9, 24).

Gender disparities and attitudes toward same-sex relationships further influenced sexual behavior frequency. Male students reported significantly higher sexual activity than females, aligning with findings from Lyu et al.’s (5) multi-province cross-sectional study in China. Research spanning from Whitley (25) exploration of gender role orientation and sexual experience to Gewirtz-Meydan et al.’s (26) analysis of gender roles in romantic intimacy consistently underscores the profound impact of sociocultural gender expectations on sexual norms. Intriguingly, students opposing same-sex relationships exhibited the highest sexual activity rates, suggesting internal conflicts between personal attitudes and behavioral patterns (11).

Human Immunodeficiency Virus prevention interventions yielded paradoxical outcomes: students actively participating in awareness campaigns reported increased sexual activity, whereas passive recipients showed reduced engagement. This dichotomy may align with Behavioral Participation Theory (BPT) and the Health Belief Model (HBM) (27). Active participants might develop more open attitudes through deepened knowledge and role internalization, whereas passive learners may retain only superficial preventive awareness without behavioral motivation shifts. Sensitivity analyses confirmed that all key findings were robust.

This study has several limitations. First, data were collected from only one of two universities directly administered by China’s Overseas Chinese Affairs Office. Furthermore, the international student cohort at the surveyed university predominantly originated from developing countries, potentially limiting generalizability to Overseas Universities students from developed nations. Second, the high proportion of “uncertain” or “unwilling to answer” responses to sensitive questions may introduce reporting bias or social desirability bias. Third, we did not measure exact age. Although the cohort comprises first-year undergraduates (predominantly 18 years old), minor age variation could not be explored and should be considered in future studies.

The findings collectively underscore that the sexual behaviors and attitudes of first-year undergraduates in multicultural Chinese overseas-oriented universities are significantly correlated with their pre-university regional and cultural backgrounds. This phenomenon is likely attributable to the fact that first-year students’ behavioral patterns and perceptions predominantly mirror their pre-college socialization processes compared to senior cohorts, thereby accentuating geographically and culturally rooted disparities. Furthermore, subsequent acculturation within university collectives precipitates intercultural dynamics that engender heterogeneous behavioral and attitudinal outcomes, reflecting the complex interplay between inherited socio-cultural norms and emergent institutional influences (28, 29).

## 5 Conclusion

In conclusion, university sexual health education should prioritize gender role education and guidance on diverse sexual orientations to foster scientifically grounded, inclusive attitudes. Culturally tailored strategies accounting for regional backgrounds and practice-oriented interventions are critical for enhancing protective behaviors. These findings highlight the unique sexual behavior patterns and determinants among overseas Chinese university freshmen, providing valuable insights for developing culturally adaptive sexual health programs and informing future research on cross-cultural sexual behavior models and prevention strategies.

## Data Availability

The original contributions presented in the study are included in the article/Supplementary material, further inquiries can be directed to the corresponding author.
